# Non-linear modelling to describe lactation curve in Gir crossbred cows

**DOI:** 10.1186/s40781-017-0128-6

**Published:** 2017-02-10

**Authors:** Yogesh C. Bangar, Med Ram Verma

**Affiliations:** 1 Network Project on Sheep (Deccani) Improvement, Mahatma Phule Krishi Vidyapeeth, Rahuri, Maharashtra 413722 India; 2 Division of Livestock Economics, Statistics and IT, Indian Veterinary Research Institute, Bareilly, Uttar Pradesh 243122 India

**Keywords:** Gir crossbred, Lactation curve, Non-linear modelling, Gamma-type function

## Abstract

**Background:**

The modelling of lactation curve provides guidelines in formulating farm managerial practices in dairy cows. The aim of the present study was to determine the suitable non-linear model which most accurately fitted to lactation curves of five lactations in 134 Gir crossbred cows reared in Research-Cum-Development Project (RCDP) on Cattle farm, MPKV (Maharashtra). Four models viz. gamma-type function, quadratic model, mixed log function and Wilmink model were fitted to each lactation separately and then compared on the basis of goodness of fit measures viz. adjusted R^2^, root mean square error (RMSE), Akaike’s Informaion Criteria (AIC) and Bayesian Information Criteria (BIC).

**Results:**

In general, highest milk yield was observed in fourth lactation whereas it was lowest in first lactation. Among the models investigated, mixed log function and gamma-type function provided best fit of the lactation curve of first and remaining lactations, respectively. Quadratic model gave least fit to lactation curve in almost all lactations. Peak yield was observed as highest and lowest in fourth and first lactation, respectively. Further, first lactation showed highest persistency but relatively higher time to achieve peak yield than other lactations.

**Conclusion:**

Lactation curve modelling using gamma-type function may be helpful to setting the management strategies at farm level, however, modelling must be optimized regularly before implementing them to enhance productivity in Gir crossbred cows.

## Background

The lactation curve in dairy cow provides graphical representation of milk production over the course of lactation. It shows that the milk production increases with high rate up to the point of peak yield and then after milk production started decreasing with lower rate [1, 2]. The shape of lactation curve could be useful in preparation of farm management strategy regarding health, breeding, nutrition and risk of potential diseases in dairy cows [3–6].

The lactation curve cannot be fitted using linear model since the curve don’t have linear trend with time. To explain the flow of milk production over the course of lactation in dairy cows, various mathematical models have been developed [7–9]. The lactation curve is influenced by several animal level and herd level factors such breed, age, parity, season of calving and managemental practices [10]. Thus, the parameters estimated from the different mathematical models may influences due to animal-level and environmental-level factors, and leads to variation in fitting to a typical lactation curve. A suitable model which can gave best fit to the lactation curve among the various models may only be efficient to predict the peak yield or phase of potential production, and to adopt the improved managemental practices.

Therefore, the aim of the present study was to determine the most appropriate non-linear model to describe lactation curves in Gir crossbred cows.

## Methods

### Data

The present study was conducted at Research-Cum-Development Project on Cattle (RCDP), Mahatma Phule Krishi Vidyapeeth (MPKV), Rahuri, Maharashtra (India). This Project maintains Gir crossbred cows which have inheritance either of Jersey, Holstein or both. The data of half-bred and triple crossbred cows were considered as whole for analysis due to sample size in the study. The animals were kept in loose housing system. The separate buyers were provided for young calves, heifers, milch cows, dry cows, pregnant cows, breeding bulls etc. The feeding practices included lucerne, berseem, cowpea, maize, jowar and oat as green fodder and jowar kadabi as dry fodder. The concentrate mixture used for feeding different categories of animals was as per their nutritional requirement. Hand and machine milking was practiced at project and cows were milked twice a day. The production details regarding monthly milk yields of 134 of crossbred cows were collected for the period of April 2012 to March 2013. The monthly milk yields obtained from monthly milk registers for first 10 months of five lactations completed by Gir crossbred cows. The data were corrected for different date of calving by correction factor.

### Statistical analysis

The descriptive statistics were obtained for monthly lactation milk yield in five lactations. One way ANOVA was used to determine if there was significant (*p* < 0.05) difference between milk yields of five lactation at each month of lactation. Pairwise comparisons between five lactation were done using by Tukey test.

Various lactation curve models, i.e., gamma-type function, quadratic model, mixed log function and Wilmink model was fitted to describe the lactation curve of Gir crossbred cows. These models fitted to average milk yields (kg/day) as follows:

Gamma-type function [7]: *Y*_*t*_ = *at* ^*b*^ *e* ^− *ct*^

Quadratic model: *Y*_*t*_ = *a* + *bt* – *ct* ^2^

Mixed Log [8]: *Y*_*t*_ = *a* + *bt* ^0.5^ + *c* log *t*

Wilmink [9]: *Y*_*t*_ = *a* + *be* ^− *kt*^ + *ct*

Where, Y_t_ is the average milk yield on the t^th^ month, a is the initial milk yield of lactation, b and c are the ascending slope parameter up to the peak yield and the descending slope parameter after peak yield, respectively. The constant k in Wilmink model was considered as 0.61 instead of 0.05 due to superiority of goodness of fit of model due to 0.61 in preliminary analysis.

The models were tested for goodness of fit (quality of prediction) using adjusted coefficient of determination (*R*_*adj*_^2^), root mean square error (RMSE), Akaike’s Informaion Criteria (AIC) and Bayesian Information Criteria (BIC). The details of goodness fir criteria used in this study are as follows.

$$ \begin{array}{cc}\kern1em {R}_{adj}^2=1-\left[\frac{\left( n-1\right)}{\left( n- p\right)}\right]\left(1-{R}^2\right)\kern1em & \kern1em  RMSE=\sqrt{\frac{ R SS}{n- p-1}}\kern1em \\ {}\kern1em  AIC= n\  \ln (RSS)+2 p\kern1em & \kern1em  BIC= n\  \ln \left(\frac{ R SS}{n}\right)+ p\  \ln ( n)\kern1em \end{array} $$

Where R^2^ = 1- (RSS/TSS), RSS and TSS represents residual and total sum of square, respectively; n and p are the number of observations (data points) and parameters in the model, respectively. ln represents natural logarithm.

The value of R_adj_^2^ close to 1 indicates satisfactory fitting due to model. A smaller value of RMSE, AIC and BIC indicates a better fit of models. Therefore, the model which has highest adjusted *R* ^*2*^ value and lowest value of RMSE, AIC and BIC value was considered most appropriate for describing lactation curve of Gir crossbred cows. Statistical analyses was done was using SAS 9.3 Version [11]. SAS software uses Gauss-Newton method for iteration and provides least squares estimates. Further, peak yield, persistency and months in milk at peak yield were calculated using gamma-type function as described by Tekerli et al. [10] as Peak yield = a * (b/c)e^‐ b^, Persistency = ‐ (b + 1) * ln(c) and Months in milk at peak yield = b/c.

## Results and discussion

The average milk yield across lactation in first five lactations in Gir crossbred cows is presented in Table 1. The highest milk yield was observed in second month of lactation in first (10.08 kg), second (13.19), third (14.92 kg), fourth (16.59 kg) and fifth (14.41 kg) lactation. One way analysis of variance revealed that there was significant (*p* < 0.05) difference between milk yields of five lactations up to four months only. Overall there was significantly higher milk yield in fourth lactation followed by third, fifth and second lactation than first lactation of Gir crossbred cows. Maximum yield in fourth lactation than other lactations was in accordance with reports of Jingar et al. [12] in Karan Fries (crossbred) cows, but contradicted with reports of Rekik et al. [13] who reported maximum milk yield in third lactation. Because of this significant difference in five lactations, the non-linear modelling of lactation curve was undertaken lactation-wise separately to describe model fitting precisely.

**Table 1:** Monthly average milk yield (kg/day) in first five lactations of Gir crossbred cows

Month	First	Second	Third	Fourth	Fifth
1	7.41 ± 0.51^a^ (49)	11.46 ± 0.71^b^ (26)	11.94 ± 0.69^b^ (24)	13.20 ± 1.07^b^ (18)	10.50 ± 1.15^ab^ (17)
2	10.08 ± 0.50^a^ (48)	13.19 ± 0.82^ab^ (26)	14.92 ± 0.76^bc^ (24)	16.59 ± 1.03^c^ (18)	14.41 ± 1.15^bc^ (17)
3	9.81 ± 0.49^a^ (48)	10.95 ± 0.83^ab^ (26)	13.29 ± 0.83^bc^ (24)	14.82 ± 0.85^c^ (18)	13.60 ± 1.15^bc^ (17)
4	9.02 ± 0.43^a^ (45)	9.82 ± 0.85^ab^ (26)	11.87 ± 0.84^ab^ (24)	12.80 ± 0.92^b^ (17)	12.28 ± 0.97^b^ (17)
5	8.34 ± 0.45 (44)	8.74 ± 0.89 (25)	10.62 ± 0.68 (23)	10.95 ± 0.79 (17)	10.16 ± 0.94 (17)
6	7.46 ± 0.38 (40)	7.38 ± 0.80 (24)	9.02 ± 0.68 (23)	9.74 ± 0.84 (17)	8.61 ± 1.03 (17)
7	7.19 ± 0.39 (37)	6.94 ± 0.92 (19)	8.11 ± 0.72 (21)	7.86 ± 0.88 (17)	7.74 ± 1.03 (16)
8	6.63 ± 0.34 (34)	6.41 ± 0.94 (15)	7.47 ± 0.68 (19)	7.21 ± 0.99 (12)	6.53 ± 1.08 (14)
9	5.64 ± 0.37 (30)	6.29 ± 0.92 (13)	6.44 ± 0.63 (18)	6.96 ± 0.61 (10)	6.94 ± 1.20 (10)
10	5.82 ± 0.35 (23)	6.01 ± 0.76 (10)	5.55 ± 0.65 (14)	6.49 ± 0.31 (9)	6.17 ± 1.02 (9)

Figure in parenthesis indicates number of observations. Different superscript (a, b, c) differ significantly (*p* < 0.05) in same row

**Fig. 1:**
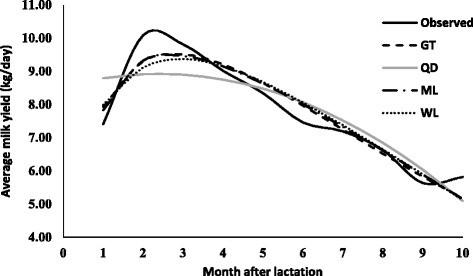
Predicted milk yield due to non-linear modeling in 1^st^ lactation of Gir Crossbred

**Fig. 2:**
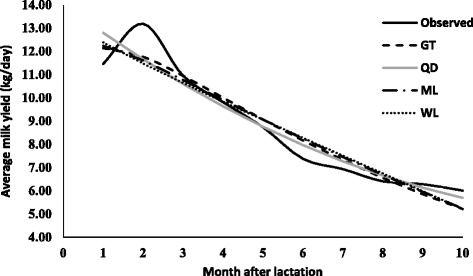
Predicted milk yield due to non-linear modeling in 2^nd^ lactation of Gir Crossbred

**Fig. 3:**
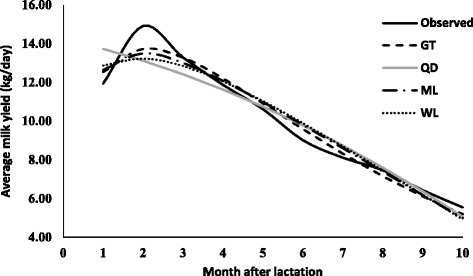
Predicted milk yield due to non-linear modeling in 3^rd^ lactation of Gir Crossbred

**Fig. 4:**
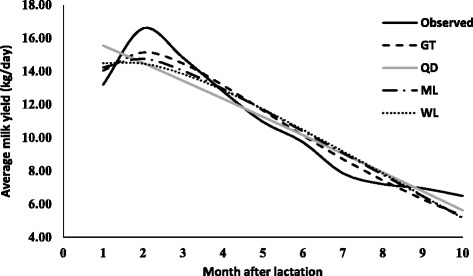
Predicted milk yield due to non-linear modeling in 4^th^ lactation of Gir Crossbred

**Fig. 5:**
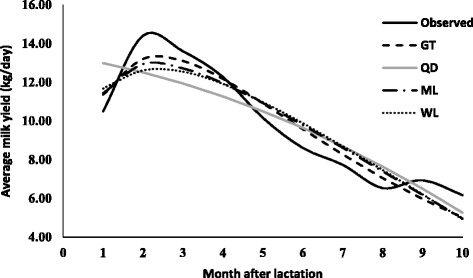
Predicted milk yield due to non-linear modeling in 5^th^ lactation of Gir Crossbred

The parameters estimates (along with standard error) due to various lactation curve models fitted average milk yield for different lactations are presented in Table 2. The graphical presentation of lactation curve for 5 lactations due to observed and predicted values is shown in Figs. 1, 2, 3, 4, and 5. In general, the estimates of initial production (parameter A) varied between five lactations and it was lowest in first lactation. This finding was similar to reports of Madalena et al. [1] in Holtein-Frisian and Holtein-Friesian × Gir cows. The parameter b and c were also found to be wide-ranging for different lactations. These findings were in accordance with reports of Boujenane [14] in Moroccan Holstein‐Friesian dairy cows. The estimates parameters due to gamma-type functions fitted to different lactations were similar to findings reported by Jingar et al. [12] in Karan Fries (crossbred) cows and Rekik et al. [13] in Holstein–Friesian cows.

**Table 2:** Lactation-wise non-linear modelling to average milk yield (kg/day) in Gir crossbred cows

LO	Model	Parameters estimates (Standard error)			Goodness of fit			
		A	b	c	*R*_*adj*_^2^	RMSE	AIC	BIC
1	GT	9.38 (0.45)	0.47 (0.10)	0.17 (0.03)	0.891	0.552	12.027	−10.091
	ML	17.53 (1.22)	−9.69 (1.44)	7.94 (1.39)	0.893	0.548	11.889	−10.229
	WL	12.93 (0.88)	−7.67 (2.03)	−0.78 (0.12)	0.850	0.650	15.310	−6.808
	QD	8.54 (1.03)	0.32 (0.43)	0.07 (0.04)	0.679	0.949	22.862	0.744
2	GT	13.83 (0.72)	0.14 (0.12)	0.13 (0.03)	0.905	0.841	20.462	−1.656
	ML	18.49 (2.05)	−6.25 (2.41)	2.82 (2.33)	0.887	0.917	22.192	0.074
	WL	12.83 (1.34)	0.60 (3.10)	−0.76 (0.18)	0.866	0.994	23.797	1.679
	QD	14.03 (0.99)	−1.27 (0.41)	−0.04 (0.04)	0.889	0.906	21.951	−0.167
3	GT	15.45 (0.59)	0.43 (0.09)	0.21 (0.02)	0.960	0.666	15.776	−6.342
	ML	26.06 (1.82)	−13.42 (2.14)	9.28 (2.07)	0.941	0.814	19.805	−2.313
	WL	17.76 (1.31)	−6.64 (3.02)	−1.28 (0.18)	0.915	0.970	23.309	1.191
	QD	14.25 (1.3)	−0.48 (0.54)	0.04 (0.05)	0.873	1.192	27.424	5.306
4	GT	17.42 (0.94)	0.42 (0.12)	0.22 (0.04)	0.929	1.029	24.491	2.373
	ML	27.97 (2.85)	−13.74 (3.36)	8.97 (3.24)	0.891	1.277	28.811	6.692
	WL	18.8 (1.94)	−5.42 (4.49)	−1.36 (0.26)	0.861	1.439	31.195	9.077
	QD	16.60 (1.72)	−1.04 (0.72)	0.01 (0.06)	0.833	1.581	33.079	10.96
5	GT	14.33 (0.95)	0.55 (0.15)	0.23 (0.04)	0.891	1.063	25.141	3.023
	ML	26.06 (2.81)	−14.62 (3.31)	10.92 (3.20)	0.847	1.258	28.507	6.389
	WL	17.86 (1.91)	−9.00 (4.41)	−1.29 (0.26)	0.807	1.415	30.856	8.738
	QD	13.37 (1.88)	−0.34 (0.79)	0.05 (0.07)	0.712	1.730	34.884	12.766

*LO* Lactation order, *GT* Gamma-type function, *QD* Quadratic model, *ML* Mixed log function, *WL* Wilmink model, *R*_*adj*_^2^ Adjusted coefficient of determination, *RMSE* Root mean square error, *AIC* Akaike’s information criteria, *BIC* Bayesian Information Criteria

In primiparous cows (first lactations), high value of adjusted R^2^ (0.679 to 0.893) indicated that non-linear modelling explained sufficient variability in shape of lactation curve, which was also reported by Boujenane [14]. However, lower estimates of adjusted coefficient of determination reported by Olori et al. [15]. The adjusted R^2^ was highest for mixed log function (0.893), followed by gamma-type function (0.891), wilmink (0.850) and least for Quadratic model (0.679). Further, The RMSE values ranged from 0.548 to 0.949 and mixed log function provided lowest value of RMSE than other models. Similarly, AIC and BIC values were also least for mixed model (11.889 and −10.229) followed by gamma-type function (12.027 and −10.091), wilmink (15.310 and −6.808) and quadratic model (22.862 and 0.744). Therefore, mixed log function was considered best model for fitting to lactation curve of primiparous Gir crossbred cows. The best fit due to mixed log function was also reported by Dongare et al. [16] while comparing gamma-type function, mixed log function and quadratic model in Sahiwal cows. The closeness of fitting between gamma-type function and mixed log function was observed, as also reported by Dongare et al. [16].

In second lactation, range of adjusted R^2^ (0.866 to 0.905) indicated better non-linear modelling in explaining the variability in lactation curve than first lactation. Gamma-type function had explained higher variation (adjusted *R* ^*2*^ = 0.905) and fitted better than other models. Further, RMSE, AIC and BIC values due to gamma-type function were lowest as 0.841, 20.462, −1.656 than that of other models. The superiority of gamma-type function for fitting of lactation curve among various models was also reported Cankaya et al. [17] in Jersey cattle. Whereas, least fitting was observed due to wilmink model with highest values of RMSE (0.994), AIC (23.797) and BIC (1.679). The trend of goodness of fit criteria for lactation curve of second lactation was in order (best first): gamma-type function < quadratic model < mixed log function < wilmink model.

Similarly, in case of third and higher lactations, highest adjusted R^2^ was observed due to gamma-type function. RMSE, AIC and BIC values due to gamma-type function were lowest for third, fourth and fifth lactations as compared to other models. The best fit due to gamma-type function model was reported by Boujenane [14] in Moroccan Holstein-Friesian dairy cows and Jingar et al. [12] in Karan Fries (crossbred) cows. The superiority in variability explained by gamma-type function in multiparous (second or more lactations) cows was in agreement with findings of previous studies [18, 19] but contrast with reports of Koçak and Ekiz [20] in Holstein cows and Dohare et al. [21] in Frieswal cows (62% Friesian and 38% Sahiwal inheritance). However, lowest adjusted *R* ^*2*^ value and highest values of RMSE, AIC and BIC were observed for quadratic model fitting, which in accordance with reports of Cilek and Keskin [22] who fitted gamma-type function, mixed log, quadratic model, cubic and exponential and polynomial regression model to lactation curve of Simmental cows. The trend of goodness of fit for third, fourth and fifth lactation was in order with best due to gamma-type function followed by mixed log function, Wilmink model and least with quadratic model.

Peak yield, Persistency, and Months in milk at peak yield due to gamma-type function are presented in Table 3. The highest peak yield was observed in fourth lactation (15.02 kg) followed by third (13.68 kg), fifth (13.35 kg), second (12.15 kg) and least in first lactation (9.45 kg). Months in milk at peak were lowest for second lactation (1.08) and highest for first lactation (2.76). However, persistency was found to be high in first lactation (2.60 months) than other lactations, which was in accordance with reports of Rekik et al. [13].

**Table 3:** Peak yield, persistency and months in milk at peak in five lactations in Gir crossbred cows

Lactation	Peak yield (kg)	Persistency (months)	Months in milk at peak yield
1	9.45	2.60	2.76
2	12.15	2.33	1.08
3	13.68	2.23	2.05
4	15.02	2.15	1.91
5	13.35	2.28	2.39

The understanding of the lactation curve of Gir crossbred cows may be an efficient tool for adopting the feeding and management practices. The gamma-type function may be used for as leading model for achieving desire productivity in Gir crossbred cows. However, the accuracy in prediction due to nonlinear models varies with variability in lactation yield in herd structure over time. Therefore, it was suggested that the optimization of lactation curve models at regular interval is necessary before their implementation.

## Conclusions

As there was significant difference in milk yield in different lactations, non-linear modelling showed varied fitting of lactation curve in first lactation and other lactations. Among the four models studied, mixed log function provided best fit of the lactation curve of primiparous cows, due to lower values of RMSE, AIC and BIC. However, in multiparous cows, gamma-type function described most appropriately the lactation curve as compared to other model. Quadratic model gave least fit to lactation curve in almost all lactations. Peak yield was highest in fourth lactation and least in first lactation. The persistency was higher in first lactation of Gir crossbred. It was suggested that lactation curve models may be helpful to setting the management strategies at farm level, however, modelling must be optimized regularly before implementing them to enhance productivity in Gir crossbred cows.

## Abbreviations

AIC: Akaike’s informaion criteria
BIC: Bayesian information criteria
p: p value
RMSE: Root mean square error
